# Prognostic Factors and a Model for Occult Breast Cancer: A Population-Based Cohort Study

**DOI:** 10.3390/jcm11226804

**Published:** 2022-11-17

**Authors:** Di Zhang, Jingtong Zhai, Lixi Li, Yun Wu, Fei Ma, Binghe Xu

**Affiliations:** Department of Medical Oncology, National Cancer Center/National Clinical Research Center for Cancer/Cancer Hospital, Chinese Academy of Medical Sciences and Peking Union Medical College, Beijing 100021, China

**Keywords:** breast, occult breast cancer, nomogram, prognosis, surveillance, epidemiology, end results database

## Abstract

Occult breast cancer (OBC) is a special type of breast cancer of an unknown primary origin. Early stage OBC is treated as stage II–III breast cancer. Currently, there are no models for predicting the survival outcomes. Hence, we aimed to evaluate the role of the positive lymph node ratio (PLNR) in OBC and further establish and validate a prognostic nomogram. Patients with stage T0N+M0 breast cancer were enrolled from the Surveillance, Epidemiology, and End Results database. Univariate and multivariate Cox analyses were used to evaluate the effects of prognostic factors on breast-cancer-specific survival (BCSS), and a nomogram was established and validated for OBC. Overall, 843 patients were included, and the 5-year BCSS rate was 92.4%. Patients with a PLNR < 0.54 had better BCSS rates than those with a PLNR ≥ 0.54. The nomogram combined clinicopathological parameters, including the PLNR, pN stage, and estrogen receptor status, and showed a higher accuracy than the TNM staging system in predicting the BCSS. The patients could be stratified into different risk groups based on their prognostic scores. Patients in the low-risk subgroup showed an improved BCSS compared those in the high-risk subgroup. In conclusion, the PLNR is an independent prognostic factor for OBC. The PLNR-based nomogram has a better predictive ability than the TNM staging system and could be of great value for the treatment of OBC and prediction of its prognosis.

## 1. Introduction

Occult breast cancer (OBC) is a sporadic tumor representing 0.1–1% of all breast cancers [1,2]. OBC manifests as axillary lymph node (LN) metastasis. However, upon clinical or imaging examinations, no primary breast tumor can be found [3,4]. OBC has traditionally been considered to be a carcinoma of unknown primary origin with a favorable prognosis and can be treated as stage II–III breast cancer [5,6,7]. The axillary LN metastasis status, estrogen receptor (ER) status, and locoregional treatment are important prognostic factors for OBC [1,2,8,9,10], with axillary LN metastasis being the strongest unfavorable prognostic factor [8,11,12].

To date, almost all studies have focused on analyzing the relationship between the LN stage and the prognosis of OBC [13]. However, according to the American Joint Committee on Cancer (AJCC) TNM staging system, the LN stage of breast cancer is only determined by the positive LN count and location (such as the inner mammary gland and supraclavicular region) [14]. The positive LN ratio (PLNR) is the ratio of the number of positive LNs to the number of regional LNs examined [15,16,17] and can reflect both the number of positive LN metastases and the quality of axillary LN dissection. Thus, it may have a greater prognostic value [18]. The PLNR has emerged as a prognostic factor for multiple cancers in several studies [19,20,21].

Thus, we aimed to evaluate the effect of the PLNR on breast-cancer-specific survival (BCSS) in women with OBC using the Surveillance, Epidemiology, and End Results (SEER) database. Since there is currently no model for predicting survival outcomes in patients with OBC, we aimed to build the first visual nomogram model so as to identify the groups at a high risk of recurrence and provide a reference for clinical diagnosis and treatment.

## 2. Materials and Methods

### 2.1. Study Population

Patients diagnosed with OBC from January 2004 to December 2015 and included in the SEER database were retrospectively reviewed. The inclusion criteria were as follows: (1) female sex; (2) pathological diagnosis of OBC; (3) age ≥ 18 years; (4) number of examined LNs (ELN) ≥ 1; and (5) number of positive LNs ≥ 1. The exclusion criteria were repeated patient identity and confirmed metastasis at the first visit. As the study used data from a public database, approval from an ethics committee was not required, as this study did not include a human or animal trial.

### 2.2. Variable Retrieval and Definition

The SEER*Stat version 8.3.4 software was used to retrieve the data. The clinicopathological variables that were collected included the age at diagnosis, tumor stage and grade (grade I–IV) according to 6th edition of the AJCC, ER status, progesterone receptor (PR) status, human epidermal growth factor receptor-2 (HER-2) status, type of therapy received (surgery, radiotherapy, and chemotherapy), number of regional nodes examined, number of positive regional nodes, survival duration, and the specific cause of death.

The BCSS was calculated from the time of the pathological diagnosis to the date of breast-cancer-related death or last follow-up. The PLNR was defined as the ratio of positive LNs to the total number of LNs removed. The histological grades were expressed as follows: grade I, differentiated; grade II, moderately differentiated; grade III, poorly differentiated; and grade IV, undifferentiated or anaplastic.

### 2.3. Statistical Analyses

Categorical data were expressed as numbers and percentages, and the chi-square test was performed to examine the differences between groups. All statistical analyses were performed using SPSS (version 23.0, Chicago, IL, USA) and R Studio software (version: 1.4.1717; RStudio, Boston, MA, USA). The X-tile software was used to determine the appropriate threshold using the minimum *p* value and maximum χ^2^. The survival curves were analyzed using the Kaplan–Meier method and the log-rank test. Multivariate Cox proportional hazard regression models were used to evaluate the prognostic factors of BCSS, and a nomogram was established. Subsequently, the discriminatory power of the nomogram model was evaluated using several methods, including the concordance index (C-index), area under the curve (AUC) values, calibration plot, and decision-making curve analysis. A two-tailed *p* value < 0.05 was considered statistically significant.

## 3. Results

### 3.1. Patients’ Characteristics

A total of 843 patients identified using the SEER database were included in the study. All the patients had histologically confirmed OBC and were in the stage T0N1–3M0. The median age at diagnosis was 59 (range: 31–98) years, and 163 (19.3%) patients had OBC of grades III and IV. The ER and PR status were positive in 56.5% and 39.6% of the cases, respectively, and HER-2 overexpression was observed in 12.1% of the patients. All the patients underwent axillary LN dissection, and more than 60% had ten or more LNs removed. Of the patients, 59.8%, 19.6%, and 20.6% were categorized as having N1, N2, and N3 diseases, respectively. Regarding the treatment modalities, 322 (38.2%) patients underwent a mastectomy, 92 (10.9%) underwent breast-conserving surgery, and the rest did not undergo breast surgery. The number of patients who received radiotherapy was roughly equal to those who did not (54.0% vs. 46.0%). Moreover, chemotherapy was administered to 75.4% of the patients. The patients’ basic characteristics are presented in Table 1.

### 3.2. Risk Factors for LN Metastasis

The relationships between LN metastasis and other clinicopathological characteristics are shown in Table 2. The pN stage was related to the number of regional ELNs and the grade. Notably, a higher pN stage was associated with a higher number of regional ELNs (χ^2^ = 70.243, *p* < 0.001). Moreover, the pN stage can influence the formulation of therapeutic strategies. Patients with a higher pN stage were more likely to receive adjuvant radiotherapy and chemotherapy. LN metastasis was not associated with age, the ER status, PR status, HER-2 status, pathological grade, or surgical modality (*p* > 0.05).

### 3.3. Prognostic Impact of PLNR on BCSS

For patients with OBC, the tumor-size-related staging is T0 by default due to the unknown location of the primary site. The LN metastasis status was regarded as an independent prognostic factor for BCSS. However, the pN stage was affected by the LN surgery method and the number of ELNs. To further explore the relationship between the LN metastasis status and OBC, the PLNR was included as a variable in the analysis. The PLNR is the ratio of the number of positive LNs to the number of detected regional LNs, simultaneously reflecting the impacts of both on prognosis.

**Figure 1:**
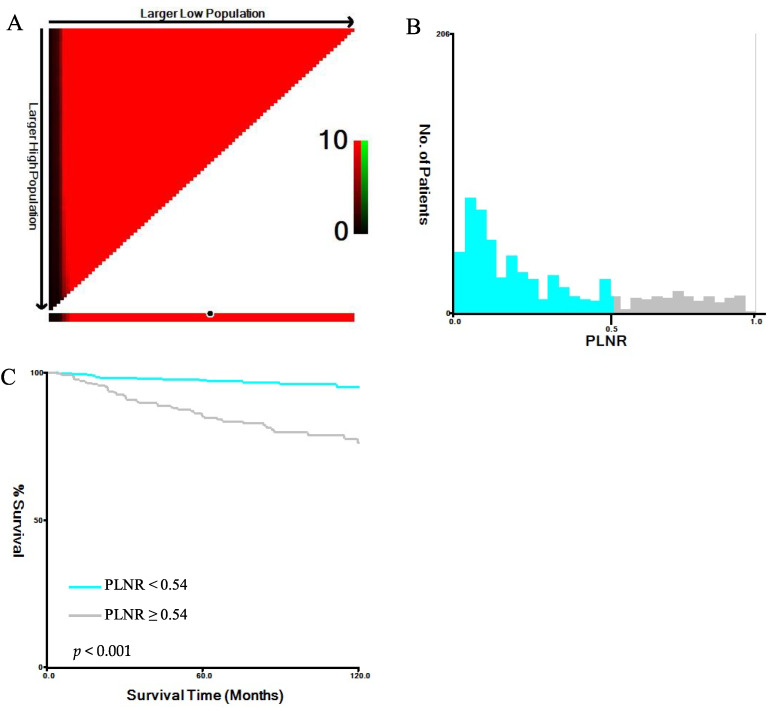
Optimal cut-off values for the PLNR. (**A**) The black dots represent the optimal cut-off values of the PLNR; (**B**) histograms of the number of patients grouped based on the optimal cut-off values of the PLNR; (**C**) Kaplan–Meier curves of patients in the PLNR < 0.54 group (blue) and the PLNR ≥ 0.54 group (grey). Abbreviation: PLNR, positive lymph node ratio.

According to the best cut-off value screened, the patients were divided into two subgroups based on the PLNR: the PLNR < 0.54 group and the PLNR ≥ 0.54 group. The corresponding maximum chi-square value was 42.3290. Among all the patients, 59.5% had a PLNR < 0.54, and 40.5% had a PLNR ≥ 0.54 (Figure 1).

### 3.4. Survival and Prognosis

**Figure 2:**
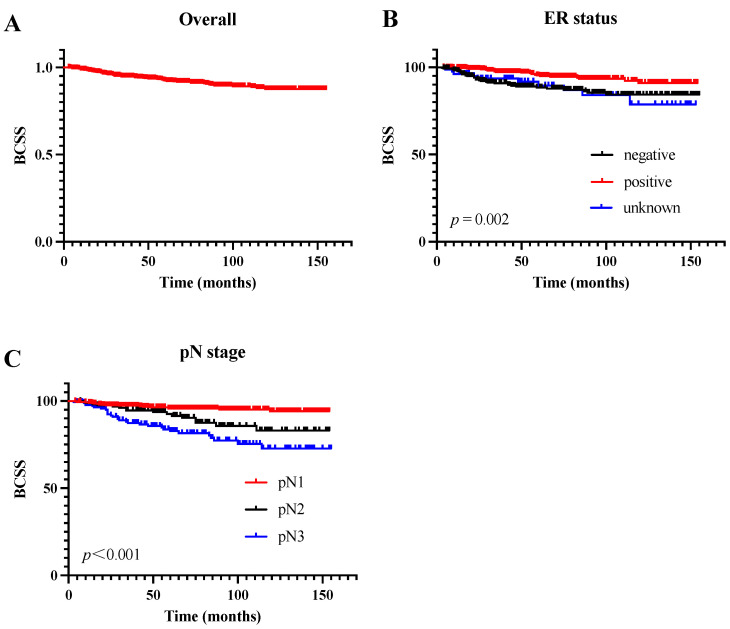
Survival curves of the cohort and the subgroups. (**A**) Kaplan–Meier curve of the cohort; (**B**) Kaplan–Meier curves of patients with different ER statuses; (**C**) Kaplan–Meier curves of patients with different pN stages. Abbreviation: ER, estrogen receptor; pN, pathological lymph node.

The median follow-up period was 68 months. Overall, the 1-, 3-, and 5-year BCSS rates of the patients were 98.7%, 94.7%, and 92.4%, respectively. The Kaplan–Meier survival curve of the overall cohort can be found in Figure 2A. The results of the univariate and multivariate Cox regression analyses for the BCSS are presented in Table 1. The univariate analysis showed that the ER status (*p* = 0.002), PR status (*p* = 0.047), and pN stage (*p* < 0.001) were correlated with the BCSS. As shown in Figure 2B, patients with a positive ER status had a better BCSS than those with a negative ER status. Additionally, the BCSS differed significantly between patients of different pN stages (Figure 2C). In the multivariate Cox regression models, the ER status, the number of regional ELNs, and the pN stage were independent prognostic factors for the BCSS. Compared with the patients with pN1 stage disease, those with pN2 and pN3 disease had a 2.6-fold and 5.4-fold respective increased risk of death (*p* = 0.005, hazard ratio (HR) = 2.584, 95% confidence interval (CI): 1.334–5.005 and *p* < 0.001, HR = 5.374, 95% CI: 3.020–9.565, respectively). Patients who had 10 or more LNs removed had a better BCSS (*p* = 0.005, HR = 0.448, 95% CI: 0.256–0.785). However, the age at diagnosis, HER-2 status, pathologic grade, postoperative radiotherapy, breast surgery, and adjuvant chemotherapy were not associated with the BCSS (*p* > 0.05).

The univariate analysis showed that a higher PLNR and pN stage were associated with a poor BCSS. In the multivariate Cox regression model of the BCSS, patients in the PLNR ≥ 0.54 subgroup had poorer prognoses than those in the PLNR < 0.54 subgroup (*p* < 0.001, HR = 3.584, 95% CI: 1.943–6.614). Moreover, compared with the patients with pN1 stage disease, the risk of breast-cancer-specific death was greater among the patients with pN2 stage (pN2 vs. pN1, *p* = 0.028, HR = 2.104, 95% CI: 1.083–4.09) and pN3 stage disease (pN3 vs. pN1, *p* = 0.002, HR = 2.662, 95% CI: 1.438–4.929). In addition, ER positivity was a protective factor against breast-cancer-specific death (*p* = 0.001, HR = 0.399). The univariate and multivariate Cox analyses of the prognostic factors are shown in Table 3.

### 3.5. Nomogram Construction and Validation

**Figure 3:**
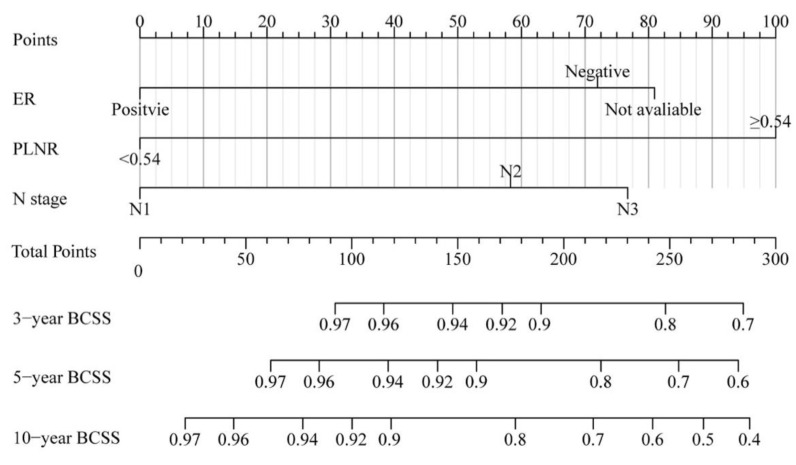
Nomogram model of BCSS in patients with OBC. Abbreviation: BCSS, breast-cancer-specific survival; OBC, occult breast cancer.

Based on the findings of the multivariate Cox analysis, a nomogram model of BCSS in patients with OBC was established, and the details are presented in Figure 3. The point for each variable is determined by drawing a vertical line from the variable to the point axis. The probability of survival at each time point is estimated by summing the total scores and positioning them on the total subscale.

**Figure 4:**
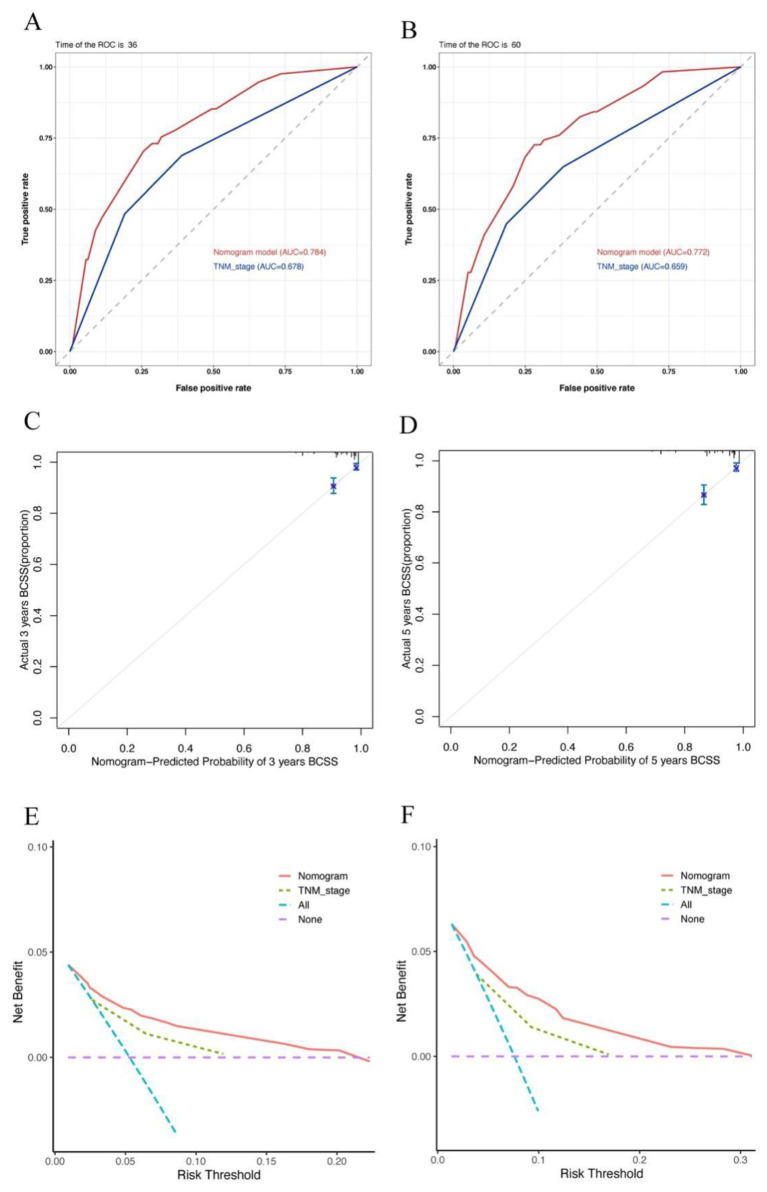
The ROC curves, calibration plots, and decision-making curve analysis of 3- and 5-year BCSS; (**A**,**B**) ROC curves for the nomogram and TNM stage; (**C**,**D**) calibration plot for the nomogram; (**E**,**F**) decision-making curve analysis of the nomogram and TNM stage. Abbreviation: ROC, receiver operator characteristic; TNM, tumor-node-metastasis.

Notably, the C-index values of our nomogram model showed a better discriminative ability than that of the TNM staging system (0.766 vs. 0.664). Similarly, the AUC values of the 3- and 5-year BCSS rates were also higher than those of the TNM staging system (3-year: 0.784 vs. 0.678, 5-year: 0.772 vs. 0.659). In addition, the calibration plots for the 3- and 5-year BCSS predictions based on the nomogram showed a satisfactory agreement between the actual and predicted clinical outcomes, further verifying the clinical value of our model. Furthermore, the decision-making curve analysis showed a good clinical net benefit. The details can be seen in Figure 4.

**Figure 5:**
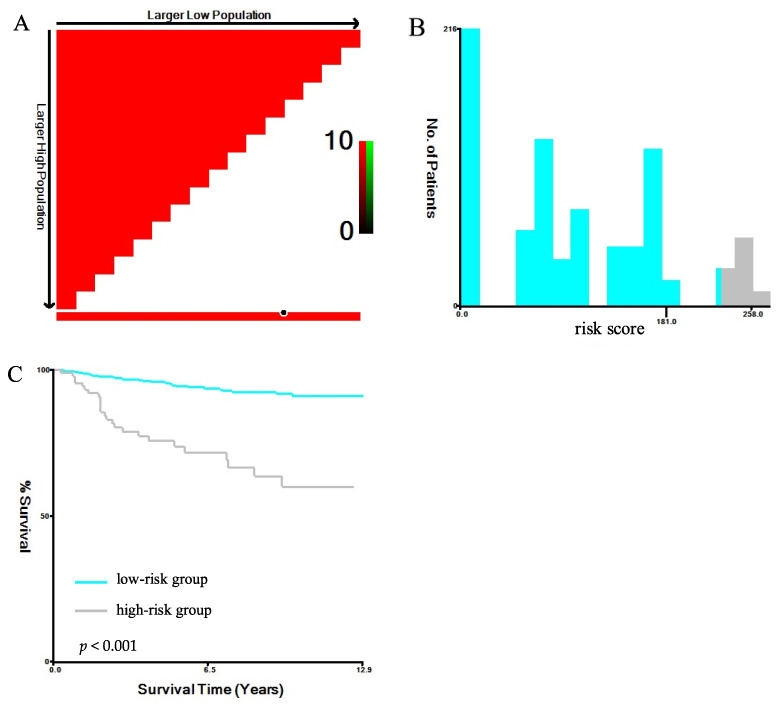
BCSS of patients among the low- and high-risk groups. (**A**) The black dots represent the optimal cut-off values of the risk scores; (**B**) histograms of the number of patients grouped based on the optimal cut-off values of the risk scores; (**C**) Kaplan–Meier curves of patients in the low-risk (blue) and high-risk (grey) groups. Abbreviation: BCSS, breast-cancer-specific survival.

Subsequently, we used X-tile software to determine the optimal cut-off value for the model-based prognostic scores. We classified the entire cohort into two new prognostic risk groups: low-risk (≤181) and high-risk (>181) (chi-square high vs. low: 59.45, relative risk: 1.00 vs. 4.92). As shown in Figure 5, compared with those in the low-risk subgroup, the BCSS of patients with OBC in the high-risk subgroups was significantly decreased (*p* < 0.001). The 3- and 5-year BCSS rates for the low- and high-risk subgroups were 96.6% vs. 78.8% and 94.6% vs. 73.7%, respectively.

## 4. Discussion

OBC is a metastatic carcinoma of the axilla with no primary breast lesions, and the T stage based on the tumor size defaults to T0. Thus, axillary LNs significantly impact the prognosis and treatment options for this particular type of breast cancer. Consistent with the existing literature [22], our study confirmed that the pN stage was associated with the survival outcomes of patients with OBC. Compared to patients with pN1 stage disease, the cancer-related risk of death was significantly increased in patients with disease of the pN2 and pN3 stages (*p* < 0.05). In addition, we also found that the ER status and the number of ELNs were significantly associated with the BCSS before including the PLNR as a prognostic variable. This is similar to a study conducted by Johnson et al., which showed that age, the pN stage, and ER status are important prognostic factors for OBC [22]. Axillary LN dissection is recommended in the National Comprehensive Cancer Network guidelines to ensure that a certain number of LNs are detected [23]. According to the AJCC staging system, no less than ten regional LNs should be considered for the accurate evaluation and staging of patients with breast cancer. The present study showed that patients with ELNs ≥10 had a better BCSS than those with ELNs <10, suggesting that an adequate number of dissected LNs is critical for patient prognosis.

Accumulating evidence suggests that the PLNR is superior to the pN stage or positive LN count as a prognostic factor for cancer patients [17,24,25]. The PLNR can reflect both the invasion of the tumor area and the effect of axillary LN dissection, theoretically providing greater prognostic value [26,27]. Therefore, our study incorporated the PLNR into the multivariate analysis as a variable for further analysis. The optimal cut-off value for the PLNR was determined in order to classify the patients into two subgroups with significant differences in survival. Patients with a PLNR ≥ 0.54 exhibited a significantly better BCSS than those with a PLNR < 0.54. In addition to the pN stage, the PLNR was also an independent prognostic factor for OBC, which implies that a certain number of LNs should be removed in cases of OBC, and this number is affected by the number of positive LNs.

To the best of our knowledge, we were the first to construct a satisfactory BCSS nomogram model for evaluating the clinical outcomes of patients with OBC. Compared with the AJCC staging system, our nomogram model was more accurate in predicting the prognosis, as confirmed by its higher C-index, better AUC values, and more consistent calibration plots. Importantly, our nomogram model can significantly stratify patient survival outcomes according to low-, intermediate-, and high-risk groups. The BCSS of these patients decreased significantly with the increasing risk class. Given the excellent predictive power of our nomogram model, we further confirmed its clinical utility.

However, our population-based study had a few limitations. Selection bias may have occurred because of the retrospective study design. In addition, some clinicopathological and therapeutic information could not be obtained, such as the HER-2 status prior to 2010, radiation dose and field, and the type of chemotherapy regimen. Despite these limitations, our established prognostic model can stratify patients with OBC into different risk groups, thus providing great value for treatment decision making and prognostic prediction.

## 5. Conclusions

In conclusion, our study comprehensively explored the clinical characteristics and survival outcomes of patients with OBC. We determined the optimal threshold for the PLNR and further evaluated its prognostic value. We also established a prognostic nomogram model of OBC and stratified the prognostic risk of patients with OBC, which is helpful in clinical decision making.

## Figures and Tables

**Table 1:** Patient characteristics.

Characteristics	Total (n = 843) N (%)	Univariable Analysis		Multivariable Analysis	
		HR (95%CI)	*p*	HR (95%CI)	*p*
Age at Diagnosis					
<60	426 (50.5)	1			
≥60	417 (49.5)	1.014 (0.626–1.645)	0.954		
ER status					
Negative	286 (33.9)	1		1	
Positive	476 (56.5)	0.398 (0.233–0.681)	0.001	0.394 (0.23–0.676)	0.001
Unknown	81 (9.6)	1.039 (0.513–2.104)	0.915	1.116 (0.544–2.288)	0.764
PR status					
Negative	410 (48.6)	1	0.047		
Positive	334 (39.6)	0.483 (0.271–0.861)	0.014		
Unknown	99 (11.7)	0.873 (0.424–1.796)	0.711		
HER-2 status					
Negative	266 (31.6)	1			
Positive	102 (12.1)	0.809 (0.299–2.194)	0.678		
Unknown	475 (56.3)	0.854 (0.474–1.537)	0.598		
Grade					
I–II	48 (5.7)	1			
III–IV	163 (19.3)	1.972 (0.586–6.641)	0.273		
Unknown	632 (75.0)	1.169 (0.363–3.77)	0.793		
pN					
N1	504 (59.8)	1		1	
N2	165 (19.6)	2.505 (1.298–4.834)	0.006	2.584 (1.334–5.005)	0.005
N3	174 (20.6)	4.765 (2.706–8.392)	<0.001	5.374 (3.02–9.565)	<0.001
Number of regional LNs examined					
<6	218 (25.9)	1		1	
≥6, <10	97 (11.5)	0.574 (0.232–1.415)	0.228	0.629 (0.249–1.594)	0.329
≥10	528 (62.6)	0.648 (0.383–1.096)	0.105	0.448 (0.256–0.785)	0.005
Breast surgery					
Mastectomy	322 (38.2)	1			
Breast-conserving surgery	92 (10.9)	1.047 (0.614–1.783)	0.867		
No	429 (50.9)	1.512 (0.719–3.178)	0.275		
Radiotherapy					
Yes	455 (54.0)	1			
No	388 (46.0)	1.241 (0.766–2.011)	0.381		
Chemotherapy					
Yes	636 (75.4)	1			
No	207 (24.6)	0.989 (0.556–1.759)	0.970		

Abbreviations: HR, hazard ratio; CI, confidence interval; ER, estrogen receptor; PR, progesterone receptor; HER-2, human epidermal growth factor receptor-2; LN, lymph node.

**Table 2:** Comparison of different N staging outcomes in occult breast cancer patients.

Characteristic	N1 (n = 504) (%)	N2 (n = 165) (%)	N3 (n = 174) (%)	χ^2^	*p*
Age at diagnosis				0.358	0.836
<60	254 (50.4)	81 (49.1)	91 (52.3)		
≥60	250 (49.6)	84 (50.9)	83 (47.7)		
ER status				6.257	0.181
Negative	157 (31.2)	62 (37.6)	67 (38.5)		
Positive	291 (57.7)	90 (54.5)	95 (54.6)		
Unknown	56 (11.1)	13 (7.9)	12 (6.9)		
PR status				8.147	0.086
Negative	231 (45.8)	84 (50.9)	95 (54.6)		
Positive	203 (40.3)	64 (38.8)	67 (38.5)		
Unknown	70 (13.9)	17 (10.3)	12 (6.9)		
HER-2 status				5.257	0.262
Negative	163 (32.3)	56 (33.9)	47 (27.0)		
Positive	53 (10.5)	21 (12.7)	28 (16.1)		
Unknown	288 (57.1)	88 (53.3)	99 (56.9)		
Grade				9.909	0.042
I–II	30 (6.0)	12 (7.3)	6 (3.4)		
III–IV	82 (16.3)	38 (23.0)	43 (24.7)		
Unknown	392 (77.8)	115 (69.7)	125 (71.8)		
Number of regional LNs examined				70.243	<0.001
1–5	172 (34.1)	15 (9.1)	31 (17.8)		
6–9	63 (12.5)	29 (17.6)	5 (2.9)		
≥10	269 (53.4)	121 (73.3)	138 (79.3)		
Breast surgery				4.508	0.342
Mastectomy	187 (37.1)	72 (43.6)	63 (36.2)		
BCS	58 (11.5)	19 (11.5)	15 (8.6)		
No	259 (51.4)	74 (44.8)	96 (55.2)		
Radiotherapy				17.914	<0.001
Yes	242 (48.0)	104 (63.0)	109 (62.6)		
No	262 (52.0)	61 (37.0)	65 (37.4)		
Chemotherapy				7.191	0.027
Yes	364 (72.2)	134 (81.2)	138 (79.3)		
No	140 (27.8)	31 (18.8)	36 (20.7)		

Abbreviations: ER, estrogen receptor; PR, progesterone receptor; HER-2, human epidermal growth factor receptor-2; BCS, breast-conserving surgery; LN, lymph node.

**Table 3:** Univariate and multivariable analyses of the prognostic factors.

Characteristics	Total (n = 843) N (%)	Univariable Analysis		Multivariable Analysis	
		HR (95% CI)	*p*	HR (95% CI)	*p*
Age at diagnosis					
<60	426 (50.5)	1			
≥60	417 (49.5)	1.014 (0.626–1.645)	0.954		
ER status					
Negative	286 (33.9)	1		1	
Positive	476 (56.5)	0.398 (0.233–0.681)	0.001	0.399 (0.233–0.684)	0.001
Unknown	81 (9.6)	1.039 (0.513–2.104)	0.915	1.123 (0.551–2.287)	0.750
PR status					
Negative	410 (48.6)	1			
Positive	334 (39.6)	0.483 (0.271–0.861)	0.014		
Unknown	99 (11.7)	0.873 (0.424–1.796)	0.711		
HER-2 status					
Negative	266 (31.6)	1	0.847		
Positive	102 (12.1)	0.809 (0.299-2.194)	0.678		
Unknown	475 (56.3)	0.854 (0.474–1.537)	0.598		
Grade					
I–II	48 (5.7)	1			
III–IV	163 (19.3)	1.972 (0.586–6.641)	0.273		
Unknown	632 (75.0)	1.169 (0.363–3.77)	0.793		
pN					
N1	504 (59.8)	1		1	
N2	165 (19.6)	2.505 (1.298–4.834)	0.006	2.104 (1.083–4.09)	0.028
N3	174 (20.6)	4.765 (2.706–8.392)	<0.001	2.662 (1.438–4.929)	0.002
Number of regional LNs examined					
<6	218 (25.9)	1			
≥6, <10	97 (11.5)	0.574 (0.232–1.415)	0.228		
≥10	528 (62.6)	0.648 (0.383–1.096)	0.105		
PLNR					
<0.50	502 (59.5)	1		1	
≥0.50	341 (40.5)	5.07 (2.887–8.905)	<0.001	3.584 (1.943–6.614)	<0.001
Breast surgery					
Mastectomy	322 (38.2)	1			
Breast-conserving surgery	92 (10.9)	1.047 (0.614–1.783)	0.867		
No	429 (50.9)	1.512 (0.719–3.178)	0.275		
Radiotherapy					
Yes	455 (54.0)	1			
No	388 (46.0)	1.241 (0.766–2.011)	0.381		
Chemotherapy					
Yes	636 (75.4)	1			
No	207 (24.6)	0.989 (0.556–1.759)	0.970		

Abbreviations: HR, hazard ratio; CI, confidence interval; ER, estrogen receptor; PR, progesterone receptor; HER-2, human epidermal growth factor receptor-2; PLNR, positive lymph node ratio; LN, lymph node.

## Data Availability

The datasets that were analyzed for this study can be found in the SEER database (https://seer.cancer.gov (accessed on 19 April 2022)).
